# Suicidal deaths in elementary school students in Korea

**DOI:** 10.1186/s13034-017-0190-3

**Published:** 2017-09-29

**Authors:** Minha Hong, Han Nah Cho, Ah Reum Kim, Hyun Ju Hong, Yong-Sil Kweon

**Affiliations:** 1 Department of Psychiatry, Myongji Hospital, Seonam University, College of Medicine, Goyang, Republic of Korea; 2 Hallym University Suicide and School Mental Health Institute, Hallym University, Anyang, Republic of Korea; 3 Department of Psychiatry, Hallym University Sacred Heart Hospital, College of Medicine, Hallym University of Korea, Anyang, Republic of Korea; 4 Department of Psychiatry, Uijeongbu St. Mary’s Hospital, College of Medicine, The Catholic University of Korea, 222 Banpo-daero, Seocho-gu, Seoul, 16591 Republic of Korea

**Keywords:** Childhood, Suicide, Death, Characteristics

## Abstract

**Background:**

The purpose of this study was to determine the characteristics of childhood suicidal deaths among elementary school students that occurred from 2011 to 2015 in Korea.

**Methods:**

The report form of each suicide case by the teacher in charge to the Education Ministry was reviewed retrospectively.

**Results:**

There were 19 suicidal deaths (12 boys, 7 girls) in elementary school students. The youngest case was a third grader (n = 1). Jumping from heights (n = 12) was the most frequently used method. Most suicides (n = 12) were committed in their homes.

**Conclusion:**

These results highlight the alarming trend of early suicidal deaths and the importance of early suicide prevention strategies, especially in schools.

**Electronic supplementary material:**

The online version of this article (doi:10.1186/s13034-017-0190-3) contains supplementary material, which is available to authorized users.

## Background

Suicide rate in Korea is the highest among the organization for economic cooperation and development (OECD) countries; therefore, child and adolescent suicide is also a major concern in public health. Additionally, suicide ranks consistently among the leading causes of death in youth globally [1]. Although there is a growing research interest in suicide in the earlier period of life [2–4], we still have insufficient knowledge about childhood suicide. It has been assumed that children are prevented from engaging in deliberate acts to take their own life, due to their limited cognitive ability to understand death and/or plan a lethal attempt. [5, 6]. On the other hand, another view is that most children acquire the concept of death and suicide by the age of 8 and are capable of planning and executing suicide [7]. According to the National Statistical Office of Korea, the reported suicide cases among children aged 5–9 years varied from 0 to 7 per year since 1983, and the average suicide rate among those aged 10–14 years was 1.1–2.3 per 100,000. In the US, suicide is the second most common cause of death among those aged 10–14 years [8]. In Austria, the average suicide rate among children aged 10–14 years was 0.72 per 100,000 from 2001 to 2014 [9]. More research focused on specific age groups from a developmental perspective should be conducted to identify variables or correlates of child and adolescent suicides.

Previously published studies including elementary school aged children were very few [10–13], if any. Groholt et al. compared risk factors and characteristics for suicide between children (< 15 years) and adolescents (15–19 years), and reported that younger suicides were less likely to be preceded by mental disorder, suicidal ideation, or precipitating stress factors [13]. Loh et al. conducted a retrospective study on suicidal deaths in those aged 10–24 years between 2000 and 2004 in Singapore, and reported that 22 suicides were among those aged 10–14 years, while 65 were among those aged 15–19 [11]. Pomili et al. evaluated suicide among children and adolescents (aged 10–17 years), and reported that suicide rate was 0.91 per 100,000, with more suicides among boys (1.21 vs. 0.59) [14]. These studies were conducted mostly with the adolescent population, and not specifically with children.

As such, children and adolescents are mostly studied together, partly because of the rarity of absolute number of child and adolescent suicides. Most research on child and adolescent suicides is conducted on clinical samples, suicide survivors, and mainly on the adolescent population, and focused on mental illness and morbidity/mortality. To our knowledge, less information on suicides among elementary school aged children in Asia is available as yet. Besides, there have been no studies to our knowledge that focus on completed suicides of elementary school students, especially from teachers’ perspectives. The studies of suicides in this age group could offer important information for prevention and interventions at schools. The purpose of this article is to present the descriptive data regarding suicidal deaths among elementary school children (aged 6/7–11/12 years) in Korea.

## Methods

According to Korea education statistics service, the rates of elementary school enrollment during 2011–2015 are 99.1, 98.6, 97.2, 96.4, and 98.5%, respectively. That is, the rate of enrollment in elementary school is virtually one hundred percent. The ministry of education in Korea recognized the seriousness of suicides and reorganized the system. One of them is developing *Student Suicide Report* form (please see Additional file 1) to assess risk factors related to school life among suicide victims. Data were collected for each case by reviewing teachers’ reports forms of suicides in elementary school. The report by the teacher in charge is mandatory for each suicide case in Korea.

This study is a retrospective review of the *Student Suicide Report* form (Additional file 1) about suicidal deaths of elementary school students in Korea between 2011 and 2015. As in other developed countries, elementary school education is compulsory for children aged 7(6)–12(11) years in Korea. The number of suicidal deaths in this study signifies the total number of suicides among the population of those aged 7–12 years in Korea. The records were examined with regard to gender, grade (at school), school life, and factors related to suicide, such as the method, site of suicide, previous experience of loss and attempts, and presence of a recognized stressful event. *Student Suicide Report* form (Additional file 1) consisted of two types of responses, one with a checklist and another one with a description. The researchers thoroughly checked both types of reports. Stressful events were extracted by the researchers from the description in the *Student Suicide Report* form. Descriptive statistical analysis was performed for all the data. This study was approved by the institutional review board of Hallym University Sacred Heart Hospital (2016-I044).

## Results

A total of 19 suicide report forms of elementary school students’ suicidal deaths during the 5 year period from 2011 to 2015 were reviewed. The majority of cases were among males (12, 63.2%). A boy in the third grade (n = 1) was the youngest case. Two cases were of fourth graders, followed by three cases of fifth graders, and 13 of sixth graders (Table 1).

**Table 1:** Characteristics of completed suicides among elementary school students between 2011 and 2015 [N (%)]

	Boys N = 12	Girls N = 7	Total N = 19
Grade			
3rd	1 (8.3)	0 (0.0)	1 (5.3)
4th	0 (0.0)	2 (28.6)	2 (10.5)
5th	1 (8.3)	2 (28.6)	3 (15.8)
6th	10 (83.3)	3 (42.8)	13 (68.4)
Residence			
Seoul	5 (41.7)	1 (14.3)	6 (31.6)
Busan	2 (16.7)	0 (0.0)	2 (10.5)
Daegu	3 (25.0)	0 (0.0)	3 (15.8)
Gyeonggi	0 (0.0)	2 (28.6)	2 (10.5)
Gangwon	0 (0.0)	1 (14.3)	1 (5.3)
Chungnam	0 (0.0)	1 (14.3)	1 (5.3)
Gyeongbuk	2 (16.7)	0 (0.0)	2 (10.5)
Jeonnam	0 (0.0)	2 (28.6)	2 (10.5)
Religion			
Christianity	3 (25.0)	1 (14.3)	4 (21.1)
Catholic	1 (8.3)	0 (0.0)	1 (5.3)
Buddhism	0 (0.0)	1 (14.3)	1 (5.3)
None	5 (41.7)	4 (57.1)	9 (47.4)
Unknown	3 (25.0)	1 (14.3)	4 (21.1)
Year of suicide			
2011	1 (8.3)	0 (0.0)	1 (5.3)
2012	2 (16.7)	1 (14.3)	3 (15.8)
2013	3 (25.0)	2 (28.6)	5 (26.3)
2014	5 (41.7)	2 (28.6)	7 (36.8)
2015	1 (8.3)	2 (28.6)	3 (15.8)
Living with both parents	9 (75.0)	5 (71.4)	14 (73.7)

**Fig. 1:**
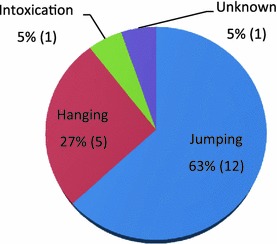
Suicide methods (n)

**Fig. 2:**
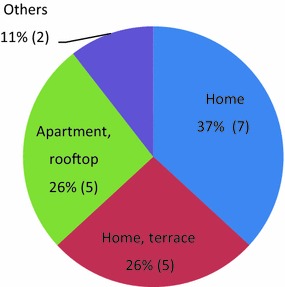
Suicide site

Of 19 suicidal deaths, jumping from a height (n = 12, 63%) was the most frequently used method (Fig. 1). Hanging (n = 5, 27%) was the second most common method of suicide, followed by intoxication (n = 1, 5%) and unknown methods (n = 1, 5%) (Fig. 1). The majority of cases occurred at home (either inside or the terrace) (n = 12, 63%) (Fig. 2).

A suicide note was found in two cases (data not shown). Any contact (with family, friend, or teacher, etc.) before suicide was found in one case (data not shown). A history of self-mutilation was found in one case and suicide attempt was found in two cases (Table 2). The previous experience of loss within a year was found in one case and any experience of suicidal event in the past was found in three cases (Table 2).

**Table 2:** Previous experience of loss and attempts

	N	%
Prior history		
None	1	5.3
Self-mutilation	1	5.3
Suicide attempt	2	10.5
Unknown	15	78.9
Recent (within a year) experience of loss		
None	3	15.8
Yes	1	21.1
Unknown	15	78.9
Experience of suicide event		
None	2	10.5
Suicide of relatives	3	15.8
Unknown	14	73.7

In terms of stressful events around suicides, teachers were reported in the existence of stressful event in four cases, and there were ten cases of such stressful events identified by researcher (Table 3).

**Table 3:** Recognized stressful events

Reported recent stressful event	By teacher	By researcher
None	15 (79.0)	9 (47.4)
Yes	4 (21.0) Peer relational problem in group activity (1) Conflict within family (1) Parent counseling request (1) Rejection (1)	10 (52.6) Scolding from parents (3) Conflict within family (2) Divorce (1) Breaking window (1) Failing in basketball game’s preliminary round (1) Rejection (1) Parent counseling request (1)

## Discussion

This study provides representative data of suicidal deaths in elementary school students in Korea, given that the rate of enrollment in elementary school is virtually one hundred percent. The number of suicidal deaths was 19 in this study, signifying the total number of suicides among those aged 7–12 years in Korea.

The findings of this study are consistent with previous reports that the majority of those who died were male [10, 15], that the gender disparity becomes more apparent with increasing age [16], and that the youngest age of the case of suicidal death was about 9 years, who was a third grader [10, 12, 17]. Two recent systematic reviews of child suicides reported that youngest ages among cases of completed suicides was 8 years [10] and 9 years [3] respectively. Dervic et al. reported on seven suicides among children aged 5–9 years using data from Statistics Austria between 1970 and 2001 [6]. In our study, the youngest case was that of a boy in the third grade, whose exact age is not determined. We can assume that he might be 8–9 years old, because in Korea, children usually enter the elementary school at the age of 6–7 years. A recent research suggested that suicidal cognitions and behaviors in early childhood (ages 3–7 years) should be taken seriously as a marker of risk for ongoing suicidal ideation/behavior [18]. Considering that the children’s understanding of suicide is related to cognitive maturity and experience [19, 20] and that the reported cases of suicidal deaths are shifting from adolescents to elementary school aged children, the populations with which suicide prevention strategies need to be conducted should be expanded.

In our study, the most common method of suicide was jumping from a height in both boys and girls. This is inconsistent with reports from other countries that hanging was the predominant method of suicide in children and young adolescents [3, 11, 21]. This is not surprising given that previous reports of greater prevalence of suicides by jumping from heights were in areas with more tall residential buildings [22], along with a high proportion of apartment residence and high population density in Korea. While the second most common method of suicide is using firearms in other countries [5, 21], it is not considered as a suicide method because they are forbidden by law in Korea. Another similar example is the reduction in suicides using pesticides after 3-year phased bans of pesticides in Sri Lanka [23]. This finding implies that strict restrictions on access to lethal means in this population can be one strategy for suicide prevention [24].

Previous studies have noted that in terms of residence, Seoul, Busan, and Daegu city are metropolitan areas, whereas Gyeonggi, Gangwon, Chungnam, Gyeongbuk, and Jeonnam are non-metropolitan areas. The completed suicides accounted for more than half (57.9%) in metropolitan areas. Although Gyeonggi is a heterogeneous area where urban and regional characteristics coexist, it is considered to be metropolitan given that it is densely populated, close to Seoul, more industrialized, and has developed traffic systems and more tall buildings and apartments. Including Gyeonggi as metropolitan area, the completed suicide rate reaches almost 70%. Our findings are not consistent with previous studies suggesting that greater urban density was generally associated with lower suicide rates in adult and elderly population [25, 26]. One possible assumption is that the high relatively more children than elderly population are living in big cities and using jumping from a height as common method of suicide might make the difference. Disparity toward metropolitan areas in children can also be interpreted in the similar context as the suicide method.

In this study, the most frequent suicide site was the home, which is compatible with the previous results [27–29]. Other findings such as previous experience of loss and attempts and recognized stressful events, however, were not consistent with those of prior studies [2, 24]. This is partly due to the data source which reflects the perspective of the teacher in charge. Especially, stressful events related to the victim recognized by teachers and by researchers differ vastly. Presence of stressful events was recognized more by researchers (52.6%) than by teachers (26.3%). As shown in Table 3, both the teacher and researcher were aware of stressful events in four cases, such as conflicts within the family (1), parent counseling request from the school (1), rejection from friend (1), and being scolded by parent (1), which were recognized as peer relational problems in group activity by the teacher. The researchers identified that there were precipitating factors such as arguments between family members or scolding from parents, divorce of parents, and failing in basketball game in six cases that were not recognized by teachers. This discrepancy of reporting between researcher and school teachers might result from a lack of understanding about child and adolescent mental health.

This study has several limitations. First, the sample size was small due to the number of completed suicide cases being 19, a common problem of single country suicide studies. Second, the study period was relatively short. Due to these limitations, we could provide only descriptive data, and not an inferential analysis. Finally, data source was mainly teachers’ report forms. Thus, variables may reflect mainly teachers’ perspectives. It is possible that school adjustment problems of students were overlooked by teachers.

## Conclusion

The findings of this study provide valuable information about elementary school aged children, especially from the teachers’ perspectives, and can be utilized in planning suicide prevention strategies at school. Further research is needed to investigate clinically significant variables and psychosocial factors using psychological autopsy.

## Additional file

**Additional file 1.** Data: Student Suicide Report Form by teachers. This includes the form on which teachers recorded details of each case of suicidal death of children in the school.

## References

[1] Organization WH (2014). Preventing suicide: a global imperative.

[2] Freuchen A, Groholt B (2015). Characteristics of suicide notes of children and young adolescents: an examination of the notes from suicide victims 15 years and younger. Clin Child Psychol Psychiatry.

[3] Dervic K, Brent DA, Oquendo MA (2008). Completed suicide in childhood. Psychiatric Clin N Am.

[4] Pfeffer CR, Conte HR, Plutchik R, Jerrett I (1979). Suicidal behavior in latency-age children: an empirical study. J Am Acad Child Psychiatry.

[5] Brent DA, Baugher M, Bridge J, Chen T, Chiappetta L (1999). Age- and sex-related risk factors for adolescent suicide. J Am Acad Child Adolesc Psychiatry.

[6] Pfeffer CR (1997). Childhood suicidal behavior. A developmental perspective. Psychiatric Clin N Am.

[7] Tishler CL, Reiss NS, Rhodes AR (2007). Suicidal behavior in children younger than twelve: a diagnostic challenge for emergency department personnel. Acad Emerg Med Off J Soc Acad Emerg Med.

[8] Leading causes of death charts. 2014. https://www.cdc.gov/injury/wisqars/leadingcauses.html. Accessed 11 July 2017.

[9] Laido Z, Voracek M, Till B, Pietschnig J, Eisenwort B, Dervic K, Sonneck G, Niederkrotenthaler T (2017). Epidemiology of suicide among children and adolescents in Austria, 2001–2014. Wien Klin Wochenschr.

[10] Beautrais AL (2001). Child and young adolescent suicide in New Zealand. Aust NZ J psychiatry.

[11] Loh C, Tai BC, Ng WY, Chia A, Chia BH (2012). Suicide in young Singaporeans aged 10–24 years between 2000 to 2004. Arch Suicide Res Off J Int Acad Suicide Res.

[12] Soole R, Kolves K, De Leo D (2015). Suicide in children: a systematic review. Arch Suicide Res Off J Int Acad Suicide Res.

[13] Groholt B, Ekeberg O, Wichstrom L, Haldorsen T (1998). Suicide among children and younger and older adolescents in Norway: a comparative study. J Am Acad Child Adolesc Psychiatry.

[14] Pompili M, Vichi M, De Leo D, Pfeffer C, Girardi P (2012). A longitudinal epidemiological comparison of suicide and other causes of death in Italian children and adolescents. Eur Child Adolesc Psychiatry.

[15] Freuchen A, Kjelsberg E, Lundervold AJ, Groholt B (2012). Differences between children and adolescents who commit suicide and their peers: a psychological autopsy of suicide victims compared to accident victims and a community sample. Child Adolesc Psychiatry Mental Health.

[16] Shaffer D, Gould MS, Fisher P, Trautman P, Moreau D, Kleinman M, Flory M (1996). Psychiatric diagnosis in child and adolescent suicide. Arch Gen Psychiatry.

[17] Groholt B, Ekeberg O (2003). Suicide in young people under 15 years: problems of classification. Nord J Psychiatry.

[18] Whalen DJ, Dixon-Gordon K, Belden AC, Barch D, Luby JL (2015). Correlates and consequences of suicidal cognitions and behaviors in children ages 3 to 7 years. J Am Acad Child Adolesc Psychiatry.

[19] Mishara BL (1999). Conceptions of death and suicide in children ages 6-12 and their implications for suicide prevention. Suicide Lifethreat Behav.

[20] Normand CL, Mishara BL (1992). The development of the concept of suicide in children. OMEGA J Death Dying.

[21] Dervic K, Friedrich E, Oquendo MA, Voracek M, Friedrich MH, Sonneck G (2006). Suicide in Austrian children and young adolescents aged 14 and younger. Eur Child Adolesc Psychiatry.

[22] Fischer EP, Comstock GW, Monk MA, Sencer DJ (1993). Characteristics of completed suicides: implications of differences among methods. Suicide Lifethreat Behav.

[23] Knipe DW, Chang SS, Dawson A, Eddleston M, Konradsen F, Metcalfe C, Gunnell D (2017). Suicide prevention through means restriction: impact of the 2008-2011 pesticide restrictions on suicide in Sri Lanka. PLoS ONE.

[24] Gould MS, Kramer RA (2001). Youth suicide prevention. Suicide Lifethreat Behav.

[25] Kim MH, Jung-Choi K, Jun HJ, Kawachi I (2010). Socioeconomic inequalities in suicidal ideation, parasuicides, and completed suicides in South Korea. Soc Sci Med.

[26] Hong J, Knapp M (2013). Geographical inequalities in suicide rates and area deprivation in South Korea. J Mental Health Policy Econ.

[27] Lee CJ, Collins KA, Burgess SE (1999). Suicide under the age of eighteen: a 10-year retrospective study. Am J Forensic Med Pathol.

[28] Uzun I, Karayel FA, Akyildiz EU, Turan AA, Toprak S, Arpak BB (2009). Suicide among children and adolescents in a province of Turkey. J Forensic Sci.

[29] Agritmis H, Yayci N, Colak B, Aksoy E (2004). Suicidal deaths in childhood and adolescence. Forensic Sci Int.

