# Supplementary material for: Suicidal deaths in elementary school students in Korea
Source: Child Adolesc Psychiatry Ment Health. 2017 Sep 29;11:53. doi: 10.1186/s13034-017-0190-3 (PMC5622440; doi:10.1186/s13034-017-0190-3)
Supplement: Supplementary file 1 — Additional file 1. Data: Student Suicide Report Form by teachers. This includes the form on which teachers recorded details of each case of suicidal death of children in the school. [file 13034_2017_190_MOESM1_ESM.docx]

**Student Suicide Report**

OO School principal <signature>

|  | Name of School | Class | Name (Sex) | Date of Birth | Parents occupation |
| --- | --- | --- | --- | --- | --- |
| Suicide |  | 0-0 | ( ) | 0000-00-00 | Father:  Mother: |

Details of the suicide

1. Time :
2. Place :
3. Process (How & Why) :

Date

Reported by:

| Special Notes on Student (with regard to school activities, peer relationship, family situations, mental/behavioral problems, etc.) |  |
| --- | --- |

| Postventions and other plans  (media & public announcement, managing the family of suicide student., postvention, etc.) |  |
| --- | --- |

྿ Check for details about the student below.

| Family issues  (Check all) | ⬜ Parents divorced ⬜ Parents separated ⬜ Father died ⬜ Mother died  ⬜ Others (e.g. Mother left home when the student was 5 years old and is not in contact until now) | |
| --- | --- | --- |
|  | Brothers and sisters | ⬜ No ⬜ Yes (No. of sons:___ and daughters: ___, Birth order among the siblings: ___) |
| Living with | ⬜ both parents ⬜ only father ⬜ only mother  ⬜ grandparents ⬜ relatives ⬜ with other people | |
| Economic status of family | ⬜ High ⬜ Middle ⬜ Low | |
|  | ⬜ Both parents employed ⬜ Both parents unemployed  ⬜ father unemployed ⬜ mother unemployed | |
| Student’s religion | ⬜ Protestant ⬜ Catholic ⬜ Buddhist ⬜ None ⬜ Others ( ) | |
| School life | Academic achievement | ⬜ High ⬜ Middle ⬜ Low |
|  | Attendance | ⬜ Good ⬜ Bad ⬜ Many sick leaves |
|  | Peer relationship | ⬜ Good ⬜ Conflicted ⬜ Withdrawn |
|  | Student Council Experience | ⬜ No ⬜ Yes (Place: e.g. Vice president) |
|  | Club activity | ⬜ No ⬜ Yes (Name: e.g. Broadcasting club) |
|  | Behavioral problems  (Check all) | ⬜ Smoking ⬜ Alcohol ⬜ Run away from home  ⬜ Stealing ⬜ School violence offender ⬜ School violence victim  ⬜ Others (e.g. Motorcycle accident, Broke school property) |
|  | Punishment records in school or legally (check all) | ⬜ Volunteering in/outside school ⬜ Special education ⬜ Suspension ⬜ Let off with a warning  ⬜ Probation  ⬜ Others (e.g. Suspended for 7 days due to school violence) |
| Personality | ⬜ Introverted ⬜ Extraverted | |
| Health status | Physical/Mental illness | ⬜ No ⬜ Yes  (Diagnosis: ) |
| School counseling | Recent serious stress event | ⬜ No ⬜ Yes  (Incidence: )  e.g. recent divorce of parents |
|  | Troubled issues  (Check all) | ⬜ Depression (Hopelessness etc.)  ⬜ Academic achievement  ⬜ Friend conflict (quarrel, end of friendship)  ⬜ Bullying/Cyber bullying  ⬜ Money extortion, Violence victim  ⬜ Participated in a violent group  ⬜ Familial conflicts (quarrel, lack of conversation etc.)  ⬜ Domestic violence ⬜ Parent’s academic pressure  ⬜ Dating problem ⬜ Appearance issues  ⬜ Porn addiction ⬜ Game/Internet addiction  ⬜ Others ( )  e.g. lack of confidence |
|  | Suicide-related History/Experience  (Check all) | ⬜ Self-harm ⬜ Suicide attempt |
|  |  | ⬜ Parent’s suicide ⬜ Sibling’s suicide  ⬜ Relative’s suicide ⬜ Friend’s suicide |

྿If the student was assessed using ‘AMPQ-Ⅱ (Adolescent Mental-Health Problem-Behavior Questionnaire) or CPSQ (Child Problem-Behavior Screening Questionnaire)’, check the items below.

| AMPQ-II or CPSQ | Results | ⬜ Normal group ⬜ Low risk group  ⬜ High risk group |
| --- | --- | --- |
|  | Experience of counseling at School Mental Health Promotion Centers (Wee-center etc.) | ⬜ No ⬜ Yes |
|  |  | ⬜ Terminated counseling due to improvement  ⬜ Referred to Psychiatrist due to aggravation  ⬜ Terminated counseling due to refusal to participate  ⬜ Loss of contact  ⬜ Etc. (e.g. Identified as high risk group and counseled once at Wee center, Discontinued due to the parent’s refusal) |
|  | Psychiatric therapy experience | ⬜ No ⬜ Yes |
